# Identification of Novel Circular RNA Targets in Key Penumbra Region of Rats After Cerebral Ischemia-Reperfusion Injury

**DOI:** 10.1007/s12031-023-02153-8

**Published:** 2023-09-09

**Authors:** Jiabei Ye, Yudong Shan, Xiaohong Zhou, Tian Tian, Weijuan Gao

**Affiliations:** 1 Hebei Key Laboratory of Chinese Medicine Research on Cardio-Cerebrovascular Disease, Hebei University of Chinese Medicine, 326 South Xinshi Road, 050091 Shijiazhuang, Hebei Province China; 2 Department of Acupuncture and Moxibustion, Hebei Province Hospital of Chinese Medicine, 050011 Shijiazhuang, Hebei China; 3 Hebei Cangzhou Hospital of Integrated Traditional and Western Medicine, Cangzhou, 061012 China

**Keywords:** Circular RNA, Cerebral ischemia-reperfusion injury, High-throughput sequencing, Penumbra, Novel targets

## Abstract

Circular RNAs (circRNAs) are abundantly and stably expressed in the brain of mammals and humans. Some circRNAs are implicated in ischemic stroke. Therefore, we aimed to detect how circRNAs change in the key penumbra area during cerebral ischemia-reperfusion (CI/R) injury. Rats were subjected to transient middle cerebral artery occlusion (tMCAO), during which the permanent blocking period was 2 h and reperfusion time was 24 or 72 h. Then modified neurologic severity score (mNSS), triphenyl tetrazolium chloride (TTC) staining and HE staining were used to exhibiting damage between rats in different groups. The penumbra regions of all rats were dissected and total RNA was further processed for high-throughput sequencing. CircRNA expression profiles were screened and bioinformatics analyses were conducted to investigate these differentially expressed circRNAs. Some of them were verified by reverse transcription-quantitative polymerase chain reaction (RT-qPCR), followed by the establishment of a circRNA-miRNA-mRNA network and the detection of their downstream molecules. A total of 99 and 98 circRNAs were differentially expressed at CI/R 24 h and CI/R 72 h, respectively. Notably, 21 circRNAs significantly changed at both reperfusion points. Three circRNAs, namely circ.7225, circ.5415, and circ.20623 were found to be associated with CI/R injury and might be preferred targets. Common downstream miR-298-5p and Bcl-3 were found to make up the circRNA-miRNA-mRNA network. Novel circRNA targets came to light in the penumbra of rats during CI/R injury and might establish the circRNA-miRNA-mRNA relationship, thus serving as potential biomarkers for ischemic stroke treatment.

## Introduction

Stroke, which is characterized by paralysis or numbness, insanity, and dystonia, is the main cause of long-term disability and death of adults around the world. Ischemic stroke has a significantly higher prevalence than hemorrhagic stroke, accounting for more than 80% in the past decade (Dichgans et al. 2019; Lloyd-Jones et al. 2010). As an acute and severe disease, ischemic stroke not only causes long‐lasting neurological impairments but also imposes enormous social and economic burdens (Chen et al. 2020; Froehler et al. 2017; Wang et al. 2018). A combination of endovascular mechanical thrombectomy and intravenous recombinant tissue plasminogen activator (IV-rtPA) has shown to be superior to IV-rtPA alone in the clinical treatment of ischemic stroke in 5 recent major randomized trials (Khandelwal et al. 2016). However, more severe brain tissue damage and neurological dysfunction may be caused by these above interventions to restore blood flow perfusion or the body’s reperfusion, which is defined as CI/R injury (Hong et al. 2018; Lin et al. 2016). As a complex and non-negligible pathophysiological process, CI/R injury is reported to occur through various mechanisms involving oxidative stress, inflammatory response, toxic effects of excitatory amino acids, intracellular calcium overload, etc., which interact with each other to eventually result in neurological deficits and have a close relationship with the prognosis of patients (Lin et al. 2016; Wu et al. 2020). Herein, novel therapeutic directions and objectives for stroke prevention and therapy should be developed.

After being observed in eukaryotic cells for decades and thought to be byproducts of RNA splicing or pathogen-specific products, circRNAs have now become a research hotspot because of their abundance, stability, and conservativeness. Different from mature messenger RNA (mRNA), circRNA is a single-stranded, covalently closed circular transcript with neither 5′ cappings nor 3′ polyadenylations. A circRNA is created from precursor mRNA (pre-mRNA) via various mechanisms during back-splicing (Ashwal-Fluss et al. 2014; Chen 2016; Memczak et al. 2013; Nahand et al. 2020). Although circRNAs cannot code for proteins, they could serve as a sponge for microRNA, interact with RNA-binding proteins, control transcription, and translate their transcripts (Jeck and Sharpless 2014). In terms of function, recent studies have shown that circRNA molecules are rich in microRNA (miRNA) binding sites and act as miRNA sponges in cells, thereby relieving the inhibition of miRNA on its target genes and increasing the expression level of target genes. This mechanism of action is known as the competitive endogenous RNA (ceRNA) mechanism. Increasing evidence suggests that circRNAs may be involved in various human diseases and could be used as biomarkers for cancers (Wei et al. 2021), diabetes (Abbaszadeh-Goudarzi et al. 2020), cardiovascular diseases (Zhang et al. 2020a, b), and so on. Their utility as biomarkers in nervous system disorders is particularly well recognized (Lu et al. 2020; Xu et al. 2021) mainly because circRNAs in the brain of mammals are extraordinarily enriched (more plentiful than in the liver, heart, lungs, and esophagus), highly conserved, and tissue-specific (Li et al. 2021; Rybak-Wolf et al. 2015). Therefore, circRNA expression profiles in this key treatable and salvageable region following ischemic stroke, called the penumbra, are worth exploring.

We profiled the expression level and dynamic variation rules of circRNAs in the pivotal penumbra zone of adult rats subjected to CI/R injury and conducted bioinformatics analysis on differentially expressed circRNAs. After 24 and 72 h of CI/R injury, the expression of 21 circRNAs was altered in the penumbra. We validated 3 circRNAs via RT-qPCR, further predicted their downstream target miRNAs and genes, and finally constructed a circRNA-miRNA-mRNA network. The results greatly enhance our understanding of the molecular mechanisms underlying ischemic stroke and reveal circRNAs as novel and prospective therapeutic targets.

## Materials and Methods

### Animal Experiments

Adult male Sprague–Dawley rats (SPF grade; weighing 230–240 g; 42–48 days) were obtained from Beijing Vital River Laboratory Animal Technology Co., Ltd, Beijing, China (License number: SCXK (Beijing) 2016-0011). For a few days before the experiments, all animals were housed in the Experimental Animal Centre of the Hebei University of Chinese Medicine, which had a temperature of 25–26 °C, a humidity of 50–70%, and lighting from 8:00 a.m. to 2:00 p.m. All rats had free access to food and water. Hebei University of Chinese Medicine’s Ethics Committee reviewed and approved all animal experiments (DWLL2020075).

All animal experiments and following procedures complied with the ARRIVE guidelines and followed the National Institutes of Health guide for the care and use of Laboratory animals (No. 8023, 1978), the U.K. Animals (Scientific Procedures) Act and associated guidelines (1986), and Directive 2010/63/EU on animal experimentation.

All rats were randomly assigned to 3 groups: sham operation group (sham, *n* = 10); tMCAO for 2 h followed by 24 h of reperfusion (CI/R 24 h, *n* = 10); and tMCAO for 2 h followed by 72 h of reperfusion (CI/R 72 h, *n* = 10).

All surgical procedures were carried out after the animals had been anesthetized with sodium pentobarbital (Merck, Darmstadt, Germany) (30 mg/kg, intraperitoneal injection). We made efforts to minimize animal suffering. CI/R injury was induced by 2 h of tMCAO. The right common and external carotid arteries (CCA and ECA) were exposed via blunt separation through a midline ventral neck incision. The right ECA was ligated and beveled to create a 2–3 mm ECA stump. Then unilateral blood flow in the middle cerebral artery (MCA) was blocked by utilizing a monofilament suture with a poly-L-lysine-coated tip (length and diameter: 5 mm and 0.36 ± 0.02 mm, respectively, Cat# 2636A3, Beijing CINONTECH CO. LTD, China). After 2 h of blocking, the suture was removed, and reperfusion was started. It was ensured that the incision was sewed up well. We performed MCAO surgeries on both the CI/R 24 h and CI/R 72 h groups, and these groups were distinguished by different reperfusion periods. Sham operation rats were subjected to the same anesthesia and vascular dissociation process, but the monofilament suture was not advanced into the carotid artery. What is noteworthy is that laser speckle imaging technology, which allows the monitoring of cortical cerebral blood flow (PeriCam PSI System, Perimed, Stockholm, Sweden), was applied at some key time points during the surgery procedure to confirm successful blood flow occlusion. During surgery and the continuous ischemia period, a heating pad maintained the rectal temperature constant at 35 ± 0.5°C. The age, weight, sex, ischemia period, and room temperature were all carefully controlled. All animals were housed individually with abundant accessible water and food after surgery.

### Behavioral Assessment

mNSS is composed of motor, sensory, beam balance, and reflex tests. The mNSS has a score range of 0 to 18. The score is higher, neurological functional impairment is more severe. These behavioral tests were performed before MCAO and after 24 or 72 h of reperfusion after MCAO by no less than 2 investigators who were not aware of the experimental groups.

### TTC Staining

First, the brain can be taken directly after anesthesia. Because the brain tissue is not fixed with paraformaldehyde, it is soft. When taking out the brain, you should be more careful to maintain the integrity of the brain. Quick freeze in the refrigerator for about 20 min for slicing. Cut one piece every 2 mm, and the first knife is at the midpoint of the line between the anterior pole of the brain and the optic cross; the second knife is at the optic chiasma; the third knife is at the funnel handle; the fourth knife is between the funnel handle and the caudal pole of the posterior leaf. The slices were placed in TTC (Cat# G3005, Solarbio Science & Technology Co., Ltd., Beijing, China) with a conventional concentration of 2% and put into a 37 ℃ incubator for 30 min, and then turned over from time to time to evenly contact the staining solution. After that, the slices were taken out and investigators could take photos.

### HE Staining

Brains from 3 randomly chosen rats were fixed in formalin, embedded in paraffin, and sectioned at 5 mm thickness. After these sections were preprocessed, HE staining was performed using the specific staining kit (Servierbio, Wuhan, China). Finally, images were taken under an inverted microscope (Leica, Germany).

### Extraction and Purification of Brain RNA

Under deep anesthesia, 4 randomly selected rats each from the above 3 groups were sacrificed, their brains were quickly removed, and the penumbra regions of the hemisphere on the surgical side were dissected as Ashwal reported previously. Finally, these samples were snap-frozen in liquid nitrogen. Before euthanasia, none of the animals displayed any negative effects.

Total RNA was extracted using the TransZol Up Plus RNA kit (Cat# ER501-01, Trans). Total RNA samples with a standard of mass ≥ 2 μg and RNA integrity number ≥ 7.0 were subjected to subsequent analysis. Using RNA Clean XP kit (Cat# A63987, Beckman Coulter, Inc. Kraemer Boulevard Brea, CA, USA) and RNase-Free DNase kit (Cat# 79254, QIAGEN, GmBH, Germany), the above qualified total RNA was purified and then further quantified for RNA-sequencing analysis.

### Library Construction and High-Throughput Sequencing of circRNAs Relevant to MCAO at Different Reperfusion Times

After extraction and purification, ribosomal RNA (rRNA) was extracted from the total RNA samples using a VAHTS Ribo-off rRNA depletion kit (Human/Mouse/Rat; Cat# N406-02, Vazyme Biotech Co. Ltd., Nanjing, China). The samples were subjected to a series of processes to construct a library using the VAHTS Universal V6 RNA-seq Library Prep kit for Illumina (Cat# NR604-02, Vazyme Biotech Co. Ltd., Nanjing, China). Then, formal sequencing was conducted on the Illumina Novoseq 6000 platform (Illumina, San Diego, CA, USA) following the paired-end procedure. The quality control specifications included data size 20G per sample and the proportion of base mass > 20 (Q20 ratio) per orientation as ≥ 85%.

### Data and Bioinformatics Analysis

After RNA-sequencing, clean reads were obtained through filtering procedures on Seqtk (https://github.com/lh3/seqtk). Next clean reads were mapped to the rat reference genome (Rnor_6.0) which was suitable for eukaryotic transcriptome sequencing data and the mapping ratio should be more than 90%.

CircRNAs were predicted by CIRI software (Gao et al. 2015) and calculated the expression by junction reads at back-splicing sites then normalized by SRPBM (Li et al. 2015). Differentially expressed genes between groups were predicted using EdgeR software. |log2 FC| >1 and *P* value < 0.05 were considered thresholds for differentially expressed circRNAs.

Gene Ontology (GO) annotations and Kyoto Encyclopedia of Genes and Genomes (KEGG) pathway analysis were conducted to explore the functions and pathways associated with the host genes of these differentially expressed circRNAs. CircRNAs that have been verified to be differently expressed, stable binding miRNAs and their downstream target genes were predicted to construct a circRNA-miRNA-mRNA interaction network based on Miranda by Cytoscape software (v3.7.2, San Diego, USA).

### Verification of Differentially Expressed Molecules by RT-qPCR

CircRNAs that were differentially expressed as well as miRNA and mRNA were verified by RT-qPCR. Total RNA samples were prepared as described above. Then, circRNAs were reverse-transcribed using the PrimeScript RT reagent kit with gDNA Eraser (Cat# RR047A, TAKARA, Japan). The above-generated cDNA samples were amplified for detection and verification with circRNA-specific primer-crossing back-splicing sites. These amplification procedures were conducted at the Applied Biosystems QuantStudio 5 machine (Thermo Fisher Scientific, United States) using SYBR Premix Ex Taq II with Tli RNaseH (Cat# RR820A, TAKARA, Japan). The internal control was β-actin. The mRNA was reverse-transcribed and amplified using the above kits and machine. And the miRNA was tested by the All-in-One miRNA qRT-PCR detection kit (Cat# QP116, GeneCopoeia, Inc., China) and CFX Connect machine (BIO-RAD, United States). The internal control for mRNA was β-actin and for miRNA was U6. The primer sequences are listed in Table 1.

**Table 1:** The primer sequences of circRNA, β-actin, rno-miR-298-5p, and Bcl-3

Gene	Primer sequences	Product length (bp)
β-actin	F: GGAGATTACTGCCCTGGCTCCTA	150
	R: GATTTCATCGTACTCCTGCTTGCTG	
circ.7225	F: GTCACTCTGGACCCTGCTGT	126
	R: TCTTTGCGGCATCTCCCAGT	
circ.20623	F: TGCAGTTGTTTCTGAAGAGCCA	150
	R: CGGCACTTGTGTTTACAGGCT	
circ. 5415	F: TGGCAGAAGACACTGAGGCA	128
	R: ATCTTGGCCGACCTTGCTCA	
rno-miR-298-5p	GAGGAGGGCTGTTCTTCCCAAA	16
Bcl-3	F: AACTGTGAGGACCTTGTTCTGCTTC	25
	R: ACCTTCGTGCCTGAGATTAAATGGG	

### Statistical Analysis

Statistical Program for Social Sciences (SPSS) v23.0 software (SPSS, Chicago, IL, USA) was used for all statistical analyses. RNA-sequencing data between 2 groups were compared by Student’s *t*-test. Values are presented as the mean ± standard deviation. Differences between more than 2 groups were assessed using one-way ANOVA, and the Tukey test was used for comparison within groups. *P*-value < 0.05 was considered to indicate statistical significance.

## Results

### Damage to the Penumbra in CI/R Injury Rats

**Fig. 1:**
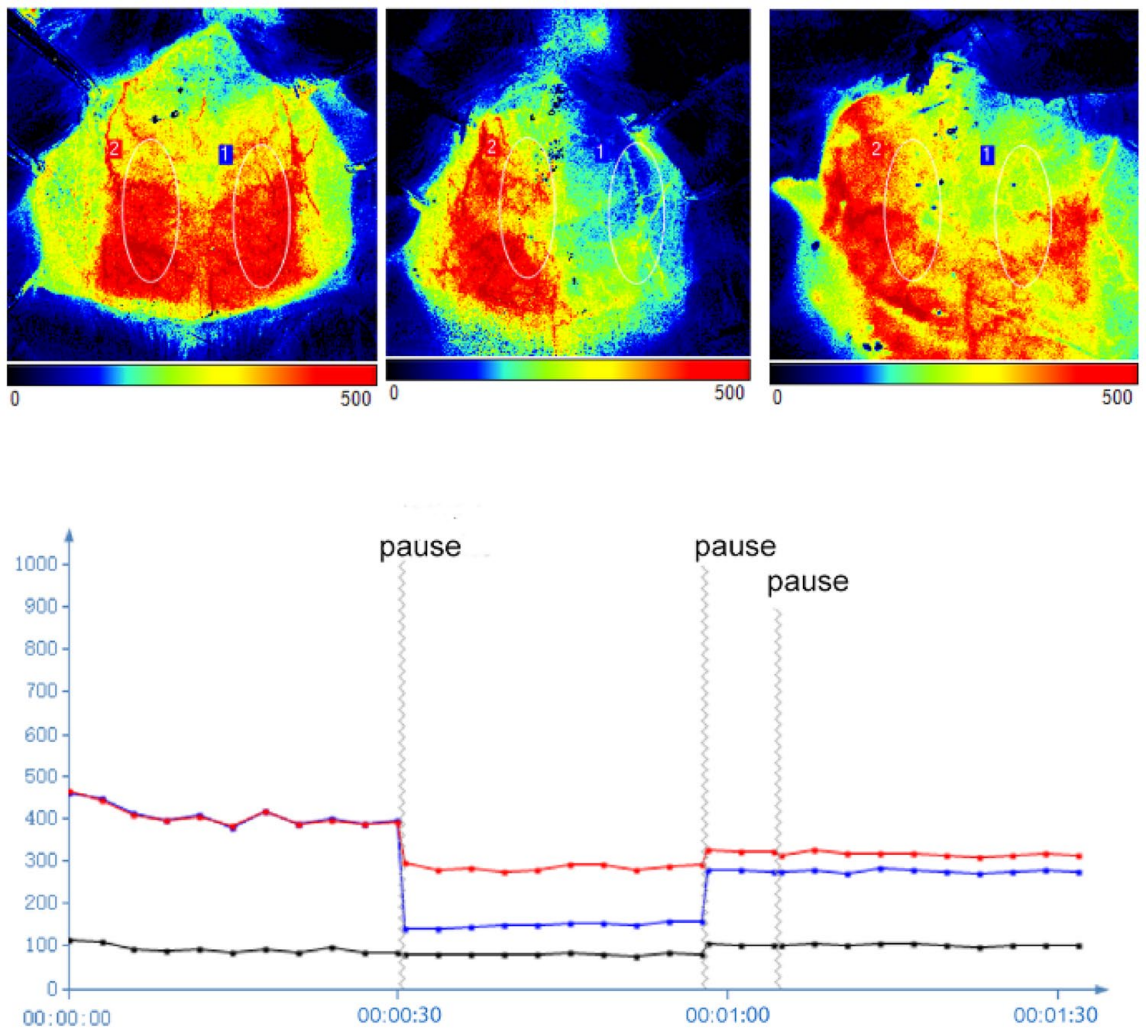
Blood flow imaging and perfusion. **a** Representative images of blood perfusion in pre-MCAO, 2h-MCAO, and post-MCAO by laser speckle imaging. **b** The line chart shows that blood flow was reduced suddenly to about 65% of pre-MCAO level on the surgical side when subjected to MCAO and recovered upon reperfusion. The blue and red lines represent perfusion on the surgical side and normal side, respectively. The black line represents average perfusion in the whole brain

**Fig. 2:**
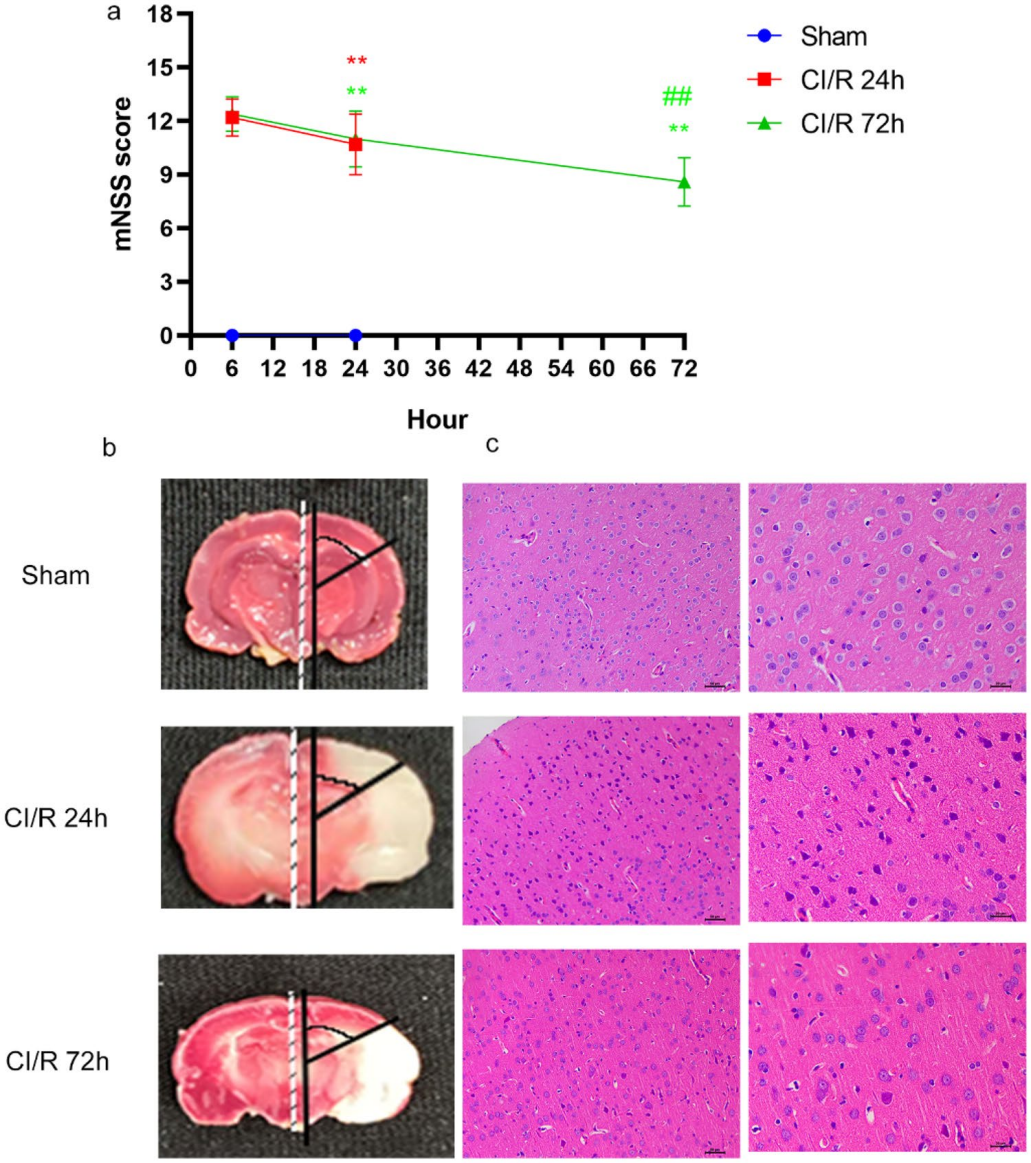
Neurological functional injury by mNSS scores (**a**). TTC staining shows the penumbra region as the ventrolateral cerebral cortex outside of the fan (**b**). HE staining of the penumbra region in different groups of rats (**c**). ^**^P < 0.01 compared with sham, ^##^*P* < 0.01 compared with CI/R 24 h, Student’s *t*-test

We induced CI/R injury in tMCAO model rats with the same period of focal ischemia but different durations of reperfusion. Blood perfusion imaging was performed to ensure that blood flow was occluded for 2 h, as shown in Fig. 1. The neurological functional injury was evaluated by mNSS scores, as shown in Fig. 2a. Figure 2b shows the dissection schematic of key penumbra regions by TTC staining. The degree of pathological damage in the penumbra decreased from CI/R 24 h to CI/R 72 h as shown by HE staining, as shown in Fig. 2c and TTC staining, as shown in Supplementary Figure.

### CircRNA Expression Profiles in the Penumbra After CI/R Injury

**Fig. 3:**
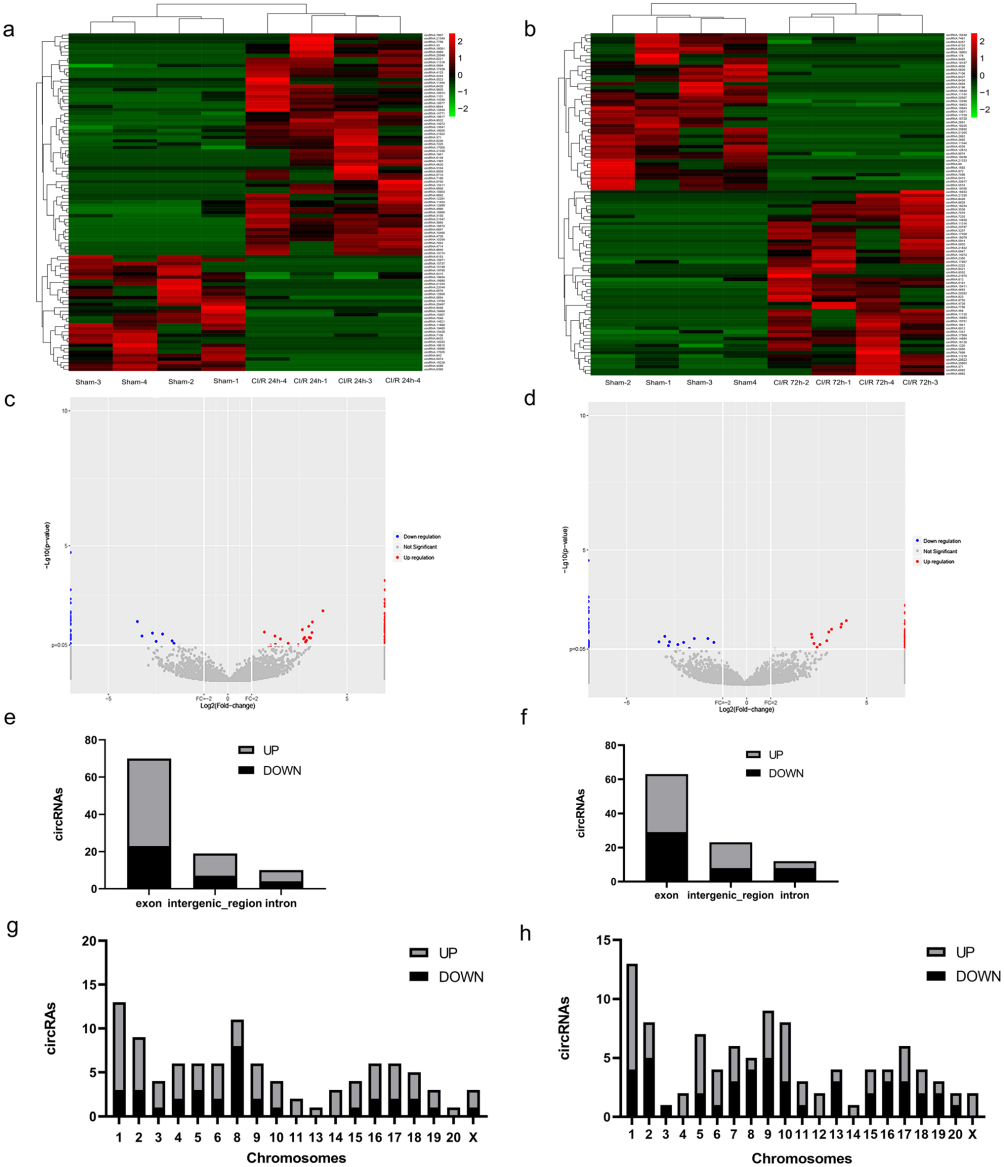
CircRNA expression profiles in the penumbra after CI/R injury. Hierarchical clustering heatmaps of differentially expressed circRNAs in the CI/R 24 h group (**a**) and CI/R 72 h group (**b**). Volcano plots exhibit differential circRNAs expression levels in the CI/R 24 h group (**c**) and CI/R 72 h group (**d**). Green or blue colors indicate downregulated circRNAs, while red color indicates upregulated circRNAs. Bar diagrams (**e** and **f**) show the location of the differentially expressed circRNAs on the rat chromosome. Bar diagrams (**g** and **h**) reveal the origin of these circRNAs. Bar diagrams reveal the characteristics of the differentially expressed circRNAs in the CI/R 24 h group (**e** and **g**) and CI/R 72 h group (**f** and **h**)

High-throughput sequencing data revealed 14374 and 13618 circRNAs at CI/R 24 h and CI/R 72 h. However, in comparison to the sham group, 99 circRNAs were found to be differentially expressed at CI/R 24 h and 98 circRNAs at CI/R 72 h. 65 circRNAs were upregulated and 34 downregulated in the CI/R 24 h group. 53 circRNAs were upregulated and 45 downregulated in the CI/R 72 h group. The hierarchical clustering heatmaps, as shown in Fig. 3a and b and volcano plots, as shown in Fig. 3c and d, revealed the differential expression of circ-RNAs. Among the markedly altered circRNAs at either CI/R 72 h or CI/R 24 h, exonic circRNAs were the most common, as shown in Fig. 3e and f. They were transcribed from almost the entire chromosomes, as shown in Fig. 3g and h.

### Summarization and Verification of Simultaneous Differentially Expressed circRNAs After Different Periods of CI/R Injury

**Fig. 4:**
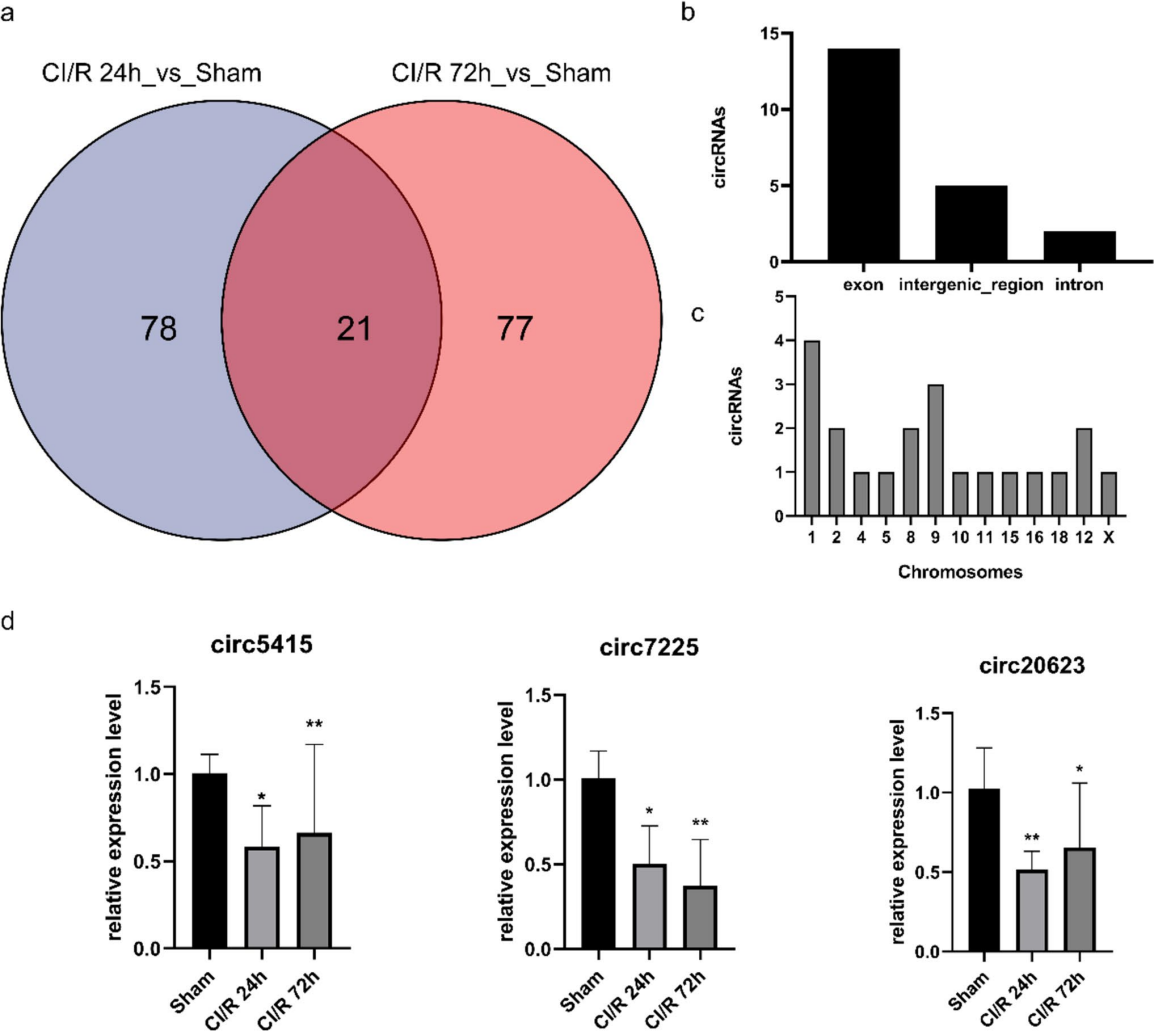
Twenty-one circRNAs were found to be differentially expressed in both CI/R 24 h and CI/R 72 h groups (**a**). The types and origins of these circRNAs were analyzed (**b** and **c**). RT-qPCR analysis showed that circ.7225, circ.20623, and circ5415 were significantly downregulated in both CI/R 24 h and 72 h groups (**d**), *P < 0.05 and **P < 0.01 compared with sham group

Given the extent of reperfusion injury varying over time, these differentially expressed circRNAs after reperfusion for 24 h or 72 h were summarized in a Venn diagram, as shown in Fig. 4a. Twenty-one circRNAs in the intersection whose expression was dramatically altered at both time points are listed in Supplementary Table. The origin and chromosomal distribution of the intersected circRNAs are shown in Fig. 4b and c, respectively. Representatives of the mentioned 21 circRNAs were then verified using RT-qPCR, as shown in Fig. 4d. Circ.7225, circ.20623, and circ.5415 were significantly downregulated in both CI/R 24 h and 72 h groups, consistent with the results of high-throughput sequencing.

### Bioinformatics Analysis of the Characteristics of the Parental Genes of Differentially Expressed circRNAs

**Fig. 5:**
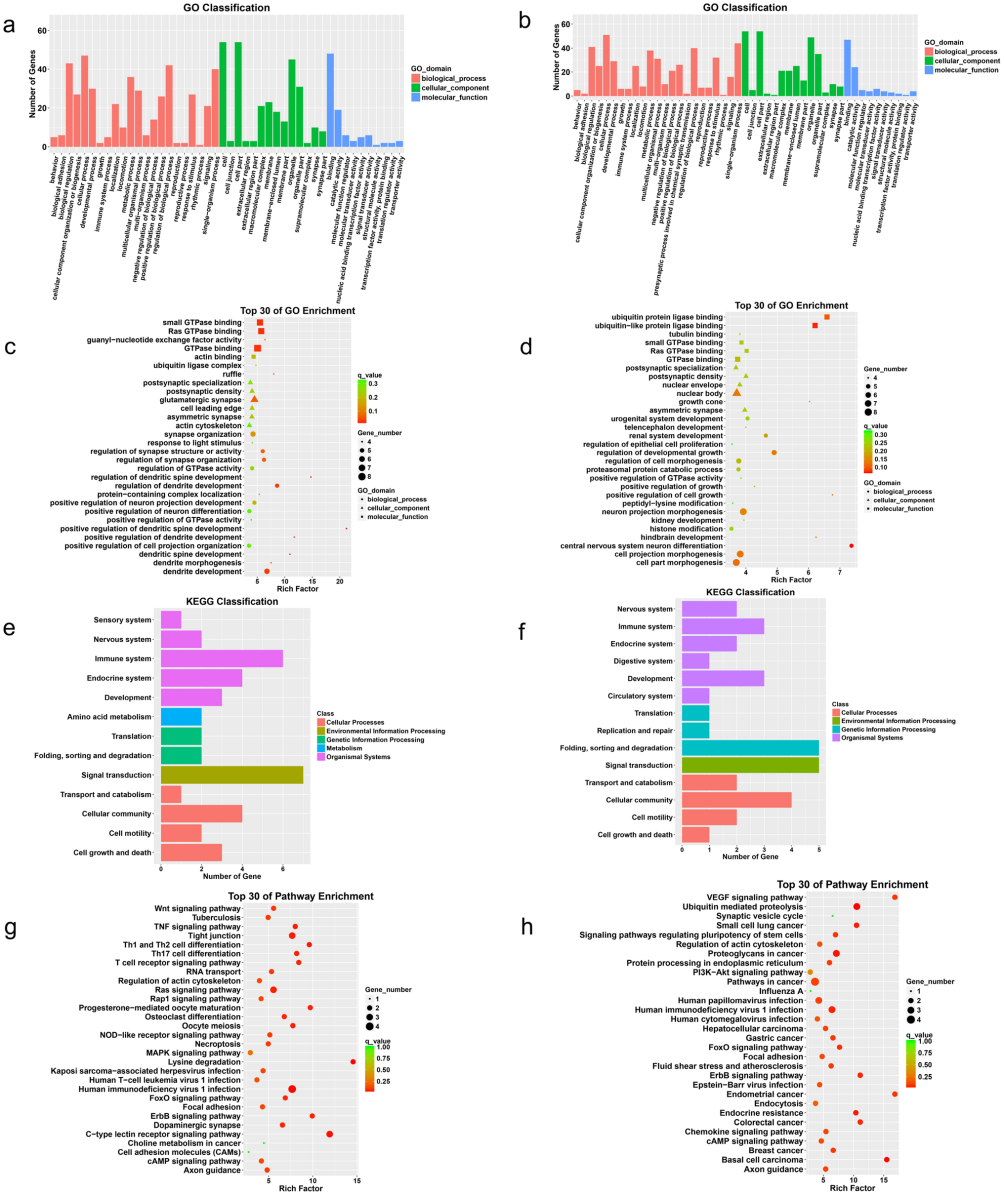
Bioinformatics analyses including GO and KEGG analysis for the characteristics and pathways of the parental genes of differentially expressed circRNAs respectively. GO histograms of the parental genes of differentially expressed circRNAs in the CI/R 24 h group (**a**) and CI/R 72 h group (**b**). The numbers of genes on the *Y*-axis are calculated according to GO terms of level 2 displayed on the *X*-axis. Bubble maps of the top 30 GO annotations in the CI/R 24 h (**c**) and CI/R 72 h (**d**) groups. **e** and **f** KEGG histograms of the parental genes of differentially expressed circRNAs in the CI/R 24 h and CI/R 72 h group respectively. KEGG pathways were classified at different levels, for example, distinguished colors revealing level 1 and the name on the vertical line signifying level 2. **g** and **h** Bubble map analyses of the top 30 KEGG pathways in the rat penumbra at CI/R 24 h and 72 h injury, respectively. In all bubble maps, the *Y*-axis represents significantly enriched GO terms or KEGG pathways and the *X*-axis denotes enriching factors on which the top 30 terms or pathways were based

GO annotation for parental genes of drastically altered circRNAs was conducted to reveal these circRNAs’ characteristics, including biological process (BP), cellular component (CC), and molecular function (MF). In the CI/R 24 h and 72 h groups, the top GO terms for BP were “cellular process, biological regulation, regulation of the biological process, and single-organism process.” Top GO terms for CC were “cell part, cell, and organelle.” Top GO terms for MF were “binding and catalytic activity,” as shown in Fig. 5a and b. Then we performed GO enrichment analysis, and the top 30 ranking predominant GO terms are displayed in Fig. 5c and d. The top enriched GO terms were about dendritic spine development (GO: 0060999; GO: 0060996; GO: 0060998) and GTPase binding (GO: 0017016; GO: 0031267; GO: 0051020) in the CI/R 24 h group then transferred into “central nervous system neuron differentiation” (GO: 0021953) and ubiquitin protein ligase binding (GO: 0044389; GO: 0031625) in the CI/R 72 h group.

### Bioinformatics Analysis for the Pathways of the Parental Genes of Differentially Expressed circRNAs

The parental genes of differentially expressed circRNAs were subjected to KEGG pathway analysis after GO annotations. From 24 to 72 h of CI/R injury, the main KEGG category was “signal transduction,” as shown in Fig. 5e and f. According to pathway enrichment analysis, we know the cAMP signaling pathway and FoxO signaling pathway were predominant, as shown in Fig. 5g and h. Except for signal transduction pathways, subordinate pathways of parental genes were related to the immune system at CI/R injury 24 h, while pathways related to cellular community and folding and sorting, and degradation were prominent at 72 h, as shown in Fig. 5e and f.

### Construction of the circRNA-miRNA-mRNA Interaction Network

**Fig. 6:**
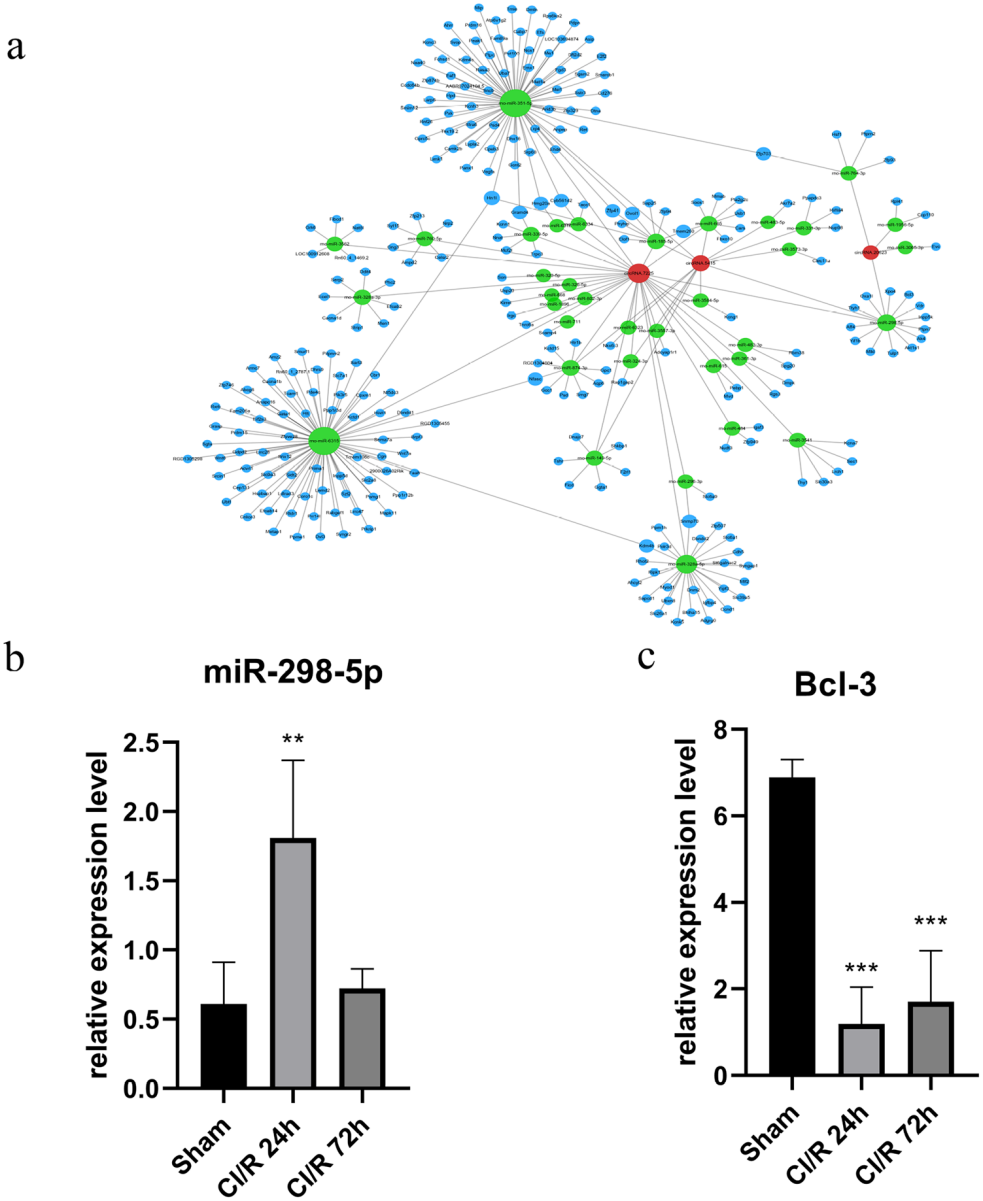
Construction of the circRNA-miRNA-mRNA interaction network. This network consisted of 3 verified circRNAs (red nodes), 36 targeted miRNAs (green nodes), and several genes (blue nodes), which depicted ceRNA mechanisms (**a**). RT-qPCR showed that miR-298-5p was significantly upregulated in the CI/R 24 h group and Bcl-3 was observably downregulated in both CI/R 24 h and 72 h groups. **P < 0.01 and ***P < 0.001 compared with sham group (**b** and **c**)

In this study, the circRNA-miRNA-mRNA interaction network was constructed to explore ceRNA mechanisms of three verified circRNAs, namely circ.7225, circ.20623, and circ.5415 with 36 predicted miRNAs and 276 predicted target mRNAs based on Miranda software. For the screening, “max energy” ≤ −64.39 was regarded as the threshold for the top 300 ceRNAs, as shown in Fig. 6a. It was noteworthy that circ.7225, circ.20623, and circ.5415 all targeted the miR-298-5p/Bcl-3 axis. Moreover, miR-298-5p was significantly upregulated at CI/R 24 h and Bcl-3 was observably downregulated at CI/R 24 h and 72 h through RT-qPCR verification, as shown in Fig. 6b and c. The primer sequences of miRNA and mRNA are also listed in Table 1. We predicted the ceRNA relationship of three novels detected circRNAs more possibly existed at CI/R 24 h.

## Discussion

We first screened differentially expressed circRNA profiles in the penumbra region of rats suffering from CI/R injury. 21 circRNAs were differentially expressed at CI/R for both 24 h and 72 h, and 3 of them were verified. Though the expression of circ.7225, circ.20623, and circ.5415 did not change with the duration of reperfusion injury, their expression level did show down-regulated during reperfusion injury. This is the first time to face whether circRNA expression level changes with the degree of reperfusion injury. Mehta found 16 circRNAs showed a persistent change from 6 to 24 h of reperfusion after transient MCAO compared with sham, but they only used the scatterplot and volcano plot to screen and real-time PCR to validate circRNAs between the sham and a 6-h reperfusion time point (Mehta et al. 2017). Lu screened 3 circRNA probes exhibiting differential expression at 5 min, 3 h, and 24 h of MCAO respectively from a mice blood sample; however, they verified other circRNAs (Lu et al. 2020). Li found that 73 circRNAs significantly altered at both 7 and 14 days after permanent distal middle cerebral artery occlusion in the non-ischemic thalamus. They could notarize the expression of 3 selected circRNAs were consistent with the high-throughput results at 14 d, but they could not be sure what happened at 7 d (Li et al. 2020). In our study, RT-qPCR validation at different injury time points could prove that the co-changed molecules would be more reliable to act as biomarkers to some extent. This is the first time to discover and fully corroborate new circRNA targets in the penumbra region after CI/R injury in rats. Further bioinformatics analysis could add more value. Many GO terms showed little differences from 24 to 72 h of CI/R injury, indicating that CI/R injury is a successional pathophysiological process. The main KEGG pathway category of “signal transduction” also verified this finding. cAMP-PKA-CREB as the typical form of cAMP signaling pathway could participate in the inhibition effect on axonal growth (Gao et al. 2020). In addition, recent studies have found that the inhibition of cAMP-specific phosphodiesterases could increase cAMP levels and finally rescue neuroinflammation in ischemic stroke (Ponsaerts et al. 2021). Ras, a kind of GTPase, responds to extracellular signals for neuronal differentiation and survival and contributes to neuroprotective signaling cascades, including MAPK, FoxO, and HIF in CI/R injury (Shi et al. 2011). These pathways were seen in KEGG analyses of parental genes associated with significantly altered circRNAs.

CeRNA is the main and classical regulator of the function of ncRNAs (Yang et al. 2020). We created the circRNA-miRNA-mRNA network to study gene expression regulation. Studies have shown that overexpression of hsa-circ-camk4 in SH-SY5Y cells significantly increased cell mortality after OGD/R, suggesting that circ-camk4 may play a key role in the progression of brain I/R injury (Zhang et al. 2020a, b). There was a shared miR-298-5p target in circRNA.7225, circ.20623, and circ.5415. It might be a useful target. Upregulation of miR-298 promoted brain injury by inhibiting the Act1/JNK/NF-κB signaling cascade as well as downstream autophagy-associated pathways following ischemic stroke (Sun et al. 2018). And the downstream target gene Bcl-3 as a nuclear member of the inhibitor of the κB family is related to brain ischemia in rats (Hu et al. 2005). Bourteele found that the alteration of NF-κB activity including Bcl-3-deficient mice could cause neuronal cell death mediated by mitochondrial apoptosis in the early stage of pathological prion protein infection (Bourteele et al. 2007). The downstream molecules of these three new targets are associated with cerebral ischemia, discovering three circRNAs more meaningful for CI/R injury. Therefore, important ceRNA relationships, circ.7225, circ.20623, and circ.5415 targeting miR-298-5p/Bcl-3, are considered major discoveries in high-throughput sequencing and bioinformatics from our study.

## Conclusion

Our study screened differentially expressed circRNAs in the vital penumbra region during CI/R injury and some hold the potential to act as biomarkers. And the ceRNA analyzed and acquired from our study may provide new potential targets and directions for mechanisms of CI/R injury. There are some limitations to this study such as rigorous clinical trials and further study to explore the mechanisms of circRNAs. In summary, the application of circRNAs is a promising strategy in the treatment of ischemic stroke.

## Data Availability

The datasets used during the present study are available from the corresponding author on reasonable request.

## References

[CR1] Abbaszadeh-Goudarzi K, Radbakhsh S, Pourhanifeh MH, Khanbabaei H, Davoodvandi A, Fathizadeh H, Sahebkar A, Shahrzad MK, Mirzaei H (2020). Circular RNA and diabetes: epigenetic regulator with diagnostic role. Curr Mol Med.

[CR2] Ashwal-Fluss R, Meyer M, Pamudurti NR, Ivanov A, Bartok O, Hanan M, Evantal N, Memczak S, Rajewsky N, Kadener S (2014). circRNA biogenesis competes with pre-mRNA splicing. Mol Cell.

[CR3] Bourteele S, Oesterle K, Weinzierl AO, Paxian S, Riemann M, Schmid RM, Planz O (2007). Alteration of NF-kappaB activity leads to mitochondrial apoptosis after infection with pathological prion protein. Cell Microbiol.

[CR4] Chen C, Liu L, Shu YQ, Jing P, Lu Y, Zhang XX, Zong XG, Guo LJ, Li CJ (2020). Blockade of HCN2 channels provides neuroprotection against ischemic injury via accelerating autophagic degradation in hippocampal neurons. Neurosci Bull.

[CR5] Chen LL (2016). The biogenesis and emerging roles of circular RNAs. Nat Rev Mol Cell Biol.

[CR6] Dichgans M, Pulit SL, Rosand J (2019). Stroke genetics: discovery, biology, and clinical applications. Lancet Neurol.

[CR7] Froehler MT, Saver JL, Zaidat OO, Jahan R, Aziz-Sultan MA, Klucznik RP, Haussen DC, Hellinger FR, Yavagal DR, Yao TL, Liebeskind DS, Jadhav AP, Gupta R, Hassan AE, Martin CO, Bozorgchami H, Kaushal R, Nogueira RG, Gandhi RH, Peterson EC, Dashti SR, Given CA, Mehta BP, Deshmukh V, Starkman S, Linfante I, McPherson SH, Kvamme P, Grobelny TJ, Hussain MS, Thacker I, Vora N, Chen PR, Monteith SJ, Ecker RD, Schirmer CM, Sauvageau E, Abou-Chebl A, Derdeyn CP, Maidan L, Badruddin A, Siddiqui AH, Dumont TM, Alhajeri A, Taqi MA, Asi K, Carpenter J, Boulos A, Jindal G, Puri AS, Chitale R, Deshaies EM, Robinson DH, Kallmes DF, Baxter BW, Jumaa MA, Sunenshine P, Majjhoo A, English JD, Suzuki S, Fessler RD, Delgado Almandoz JE, Martin JC, Mueller-Kronast NH, Investigators S (2017). Interhospital transfer before thrombectomy is associated with delayed treatment and worse outcome in the STRATIS Registry (Systematic Evaluation of Patients Treated With Neurothrombectomy Devices for Acute Ischemic Stroke). Circulation.

[CR8] Gao X, Zhang X, Cui L, Chen R, Zhang C, Xue J, Zhang L, He W, Li J, Wei S, Wei M, Cui H (2020). Ginsenoside Rb1 promotes motor functional recovery and axonal regeneration in post-stroke mice through cAMP/PKA/CREB signaling pathway. Brain Res Bull.

[CR9] Gao Y, Wang J, Zhao F (2015). CIRI: an efficient and unbiased algorithm for de novo circular RNA identification. Genome Biol.

[CR10] Hong P, Li FX, Gu RN, Fang YY, Lai LY, Wang YW, Tao T, Xu SY, You ZJ, Zhang HF (2018). Inhibition of NLRP3 inflammasome ameliorates cerebral ischemia-reperfusion injury in diabetic mice. Neural Plast.

[CR11] Hu X, Nesic-Taylor O, Qiu J, Rea HC, Fabian R, Rassin DK, Perez-Polo JR (2005). Activation of nuclear factor-kappaB signaling pathway by interleukin-1 after hypoxia/ischemia in neonatal rat hippocampus and cortex. J Neurochem.

[CR12] Jeck WR, Sharpless NE (2014). Detecting and characterizing circular RNAs. Nat Biotechnol.

[CR13] Khandelwal P, Yavagal DR, Sacco RL (2016). Acute ischemic stroke intervention. J Am Coll Cardiol.

[CR14] Lin L, Wang X, Yu Z (2016) Ischemia-reperfusion injury in the brain: mechanisms and potential therapeutic strategies. Biochem Pharmacol (Los Angel) 5(4). 10.4172/2167-0501.1000213 10.4172/2167-0501.1000213 PMC5991620 29888120

[CR15] Li F, Li C, Li X, Li Y, Zhong Y, Ling L (2020). Altered circular RNA expression profiles in the non-ischemic thalamus in focal cortical infarction mice. Aging (Albany NY).

[CR16] Li ML, Wang W, Jin ZB (2021). Circular RNAs in the central nervous system. Front Mol Biosci.

[CR17] Li Y, Zheng Q, Bao C, Li S, Guo W, Zhao J, Chen D, Gu J, He X, Huang S (2015). Circular RNA is enriched and stable in exosomes: a promising biomarker for cancer diagnosis. Cell Res.

[CR18] Lloyd-Jones D, Adams RJ, Brown TM, Carnethon M, Dai S, De Simone G, Ferguson TB, Ford E, Furie K, Gillespie C, Go A, Greenlund K, Haase N, Hailpern S, Ho PM, Howard V, Kissela B, Kittner S, Lackland D, Lisabeth L, Marelli A, McDermott MM, Meigs J, Mozaffarian D, Mussolino M, Nichol G, Roger VL, Rosamond W, Sacco R, Sorlie P, Stafford R, Thom T, Wasserthiel-Smoller S, Wong ND, Wylie-Rosett J, American Heart Association Statistics C, and Stroke Statistics S (2010). Executive summary: heart disease and stroke statistics–2010 update: a report from the American Heart Association. Circulation.

[CR19] Lu D, Ho ES, Mai H, Zang J, Liu Y, Li Y, Yang B, Ding Y, Tsang CK, Xu A (2020). Identification of blood circular RNAs as potential biomarkers for acute ischemic stroke. Front Neurosci.

[CR20] Mehta SL, Pandi G, Vemuganti R (2017). Circular RNA expression profiles alter significantly in mouse brain after transient focal ischemia. Stroke.

[CR21] Memczak S, Jens M, Elefsinioti A, Torti F, Krueger J, Rybak A, Maier L, Mackowiak SD, Gregersen LH, Munschauer M, Loewer A, Ziebold U, Landthaler M, Kocks C, le Noble F, Rajewsky N (2013). Circular RNAs are a large class of animal RNAs with regulatory potency. Nature.

[CR22] Nahand JS, Jamshidi S, Hamblin MR, Mahjoubin-Tehran M, Vosough M, Jamali M, Khatami A, Moghoofei M, Baghi HB, Mirzaei H (2020). Circular RNAs: new epigenetic signatures in viral infections. Front Microbiol.

[CR23] Ponsaerts L, Alders L, Schepers M, de Oliveira RMW, Prickaerts J, Vanmierlo T, Bronckaers A (2021) Neuroinflammation in ischemic stroke: inhibition of cAMP-specific phosphodiesterases (PDEs) to the rescue. Biomedicines 9(7). 10.3390/biomedicines9070703 10.3390/biomedicines9070703 PMC8301462 34206420

[CR24] Rybak-Wolf A, Stottmeister C, Glazar P, Jens M, Pino N, Giusti S, Hanan M, Behm M, Bartok O, Ashwal-Fluss R, Herzog M, Schreyer L, Papavasileiou P, Ivanov A, Ohman M, Refojo D, Kadener S, Rajewsky N (2015). Circular RNAs in the mammalian brain are highly abundant, conserved, and dynamically expressed. Mol Cell.

[CR25] Shi GX, Andres DA, Cai W (2011). Ras family small GTPase-mediated neuroprotective signaling in stroke. Cent Nerv Syst Agents Med Chem.

[CR26] Sun H, Zhong D, Wang C, Sun Y, Zhao J, Li G (2018). MiR-298 exacerbates ischemia/reperfusion injury following ischemic stroke by targeting Act1. Cell Physiol Biochem.

[CR27] Wang X, Xuan W, Zhu ZY, Li Y, Zhu H, Zhu L, Fu DY, Yang LQ, Li PY, Yu WF (2018). The evolving role of neuro-immune interaction in brain repair after cerebral ischemic stroke. CNS Neurosci Ther.

[CR28] Wei G, Zhu J, Hu HB, Liu JQ (2021). Circular RNAs: promising biomarkers for cancer diagnosis and prognosis. Gene.

[CR29] Wu L, Xiong X, Wu X, Ye Y, Jian Z, Zhi Z, Gu L (2020). Targeting oxidative stress and inflammation to prevent ischemia-reperfusion injury. Front Mol Neurosci.

[CR30] Xu L, Ye X, Zhong J, Chen YY, Wang LL (2021). New insight of circular RNAs’ roles in central nervous system post-traumatic injury. Front Neurosci.

[CR31] Yang W, Bai C, Zhang L, Li Z, Tian Y, Yang Z, Wang L, Wu W (2020). Correlation between serum circRNA and thyroid micropapillary carcinoma with cervical lymph node metastasis. Medicine (Baltimore).

[CR32] Zhang L, Zhang Y, Wang Y, Zhao Y, Ding H, Li P (2020). Circular RNAs: functions and clinical significance in cardiovascular disease. Front Cell Dev Biol.

[CR33] Zhang ZH, Wang YR, Li F, Liu XL, Zhang H, Zhu ZZ, Huang H, Xu XH (2020). Circ-camk4 involved in cerebral ischemia/reperfusion induced neuronal injury. Sci Rep.

